# Cytokeratin-8 in Anaplastic Thyroid Carcinoma: More Than a Simple Structural Cytoskeletal Protein

**DOI:** 10.3390/ijms19020577

**Published:** 2018-02-14

**Authors:** Dehuang Guo, Qinqin Xu, Sarabjot Pabla, John Koomen, Paul Biddinger, Ashok Sharma, Simarjot Pabla, Rafal Pacholczyk, Chien-Chung Chang, Kevin Friedrich, Kamran Mohammed, Robert C. Smallridge, John A. Copland, Jin-Xiong She, Paul M. Weinberger

**Affiliations:** 1 Departments of Otolaryngology Head/Neck Surgery, Augusta University, Augusta, GA 30912, USA; dehuang.guo@yahoo.com (D.G.); kmshafi91@gmail.com (K.M.); 2 Center for Biotechnology and Genomic Medicine, Augusta University, Augusta, GA 30912, USA; pablasarabjot@gmail.com (S.P.); assharma@augusta.edu (A.S.); pablasimarjot@gmail.com (S.P.); rpacholczyk@augusta.edu (R.P.); jshe@augusta.edu (J.-X.S.); 3 Departments of Otolaryngology, Molecular and Cellular Physiology, Feist-Weiller Cancer Center, LSU Health Shreveport, 1501 Kings Highway, Shreveport, LA 71103, USA xuqzju@gmail.com; 4 Proteomics Core Facility, H. Lee Moffitt Cancer Center, Tampa, FL 33612, USA; john.koomen@moffitt.org; 5 Departments of Pathology, Augusta University, Augusta, GA 30912, USA; pbiddinger@augusta.edu; 6 Institute of Molecular and Cellular Biology, Department of Life Science, National Tsing Hua University, Hsinchu 300, Taiwan; ccchang@life.nthu.edu.tw; 7 Medical College of Georgia, Augusta University, Augusta, GA 30912, USA; kfriedrich@augusta.edu; 8 Division of Endocrinology and Metabolism, Mayo Clinic, Jacksonville, FL 32224, USA; smallridge.robert@mayo.edu; 9 Department of Cancer Biology, Mayo Clinic, Jacksonville, FL 32224, USA; copland.john@mayo.edu

**Keywords:** cytokeratin-8, anaplastic thyroid carcinoma, apoptosis

## Abstract

Anaplastic thyroid carcinoma (ATC) is almost universally fatal. Elevated keratin-8 (KRT8) protein expression is an established diagnostic cancer biomarker in several epithelial cancers (but not ATC). Several keratins, including KRT8, have been suggested to have a role in cell biology beyond that of structural cytoskeletal proteins. Here, we provide evidence that KRT8 plays a direct role in the growth of ATCs. Genomic and transcriptomic analysis of >5000 patients demonstrates that *KRT8* mutation and copy number amplification are frequently evident in epithelial-derived cancers. Carcinomas arising from diverse tissues exhibit *KRT8* mRNA and protein overexpression when compared to normal tissue levels. Similarly, in a panel of patient-derived ATC cell lines and patient tumors, KRT8 expression shows a similar pattern. sh-RNA-mediated *KRT8* knockdown in these cell lines increases apoptosis, whereas forced overexpression of KRT8 confers resistance to apoptosis under peroxide-induced cell stress conditions. We further show that KRT8 protein binds to annexin A2, a protein known to mediate apoptosis as well as the redox pathway.

## 1. Introduction

Most cases of thyroid cancer have >95% five-year survival rate, but patients with anaplastic thyroid carcinoma (ATC) have a median survival time of less than 6 months [1,2]. While ATC is a rare disease (2% of all thyroid cancers), it represents 30–45% of thyroid cancer deaths [2,3]. Current treatment modalities fail to significantly prolong survival [2,3]; indeed, long-term survivors are so rare that they warrant case reports even on single individuals [4].

Keratins are members of the intermediate filament family of proteins [5] that have been studied in ATC and differentiated thyroid cancers as diagnostic biomarkers [6]. These proteins are traditionally understood as structural constituents, important for epithelial cell cytoskeleton structure and function. However, other proteins first classified as structural (e.g., β-catenin [7], actin [8], lens crystallins [9], and tubulin [10], among others) subsequently have been discovered to possess important non-mechanical roles (reviewed in [11]). Similarly, isolated data suggested that non-mechanical functions for keratins are accumulating. For example, keratin-17 has been shown to affect cell cycle control through binding and cytoplasmic transport of p21 [12], and may play a role in regulating the expression of multiple chemokine receptors in epithelial cancers [13]. Keratin-8 (KRT8) specifically has been associated with the multiple drug resistance (MDR) phenotype in breast cancer cells [14], and has been suggested to potentially modulate apoptosis resistance in nasopharyngeal carcinoma [15]. Despite these isolated reports, a molecular basis for a potential non-mechanical function of KRT8 has not yet been determined.

In this study, we used a bioinformatics approach to query several large-scale genomics datasets for potential alterations in *KRT8* mRNA expression, for keratin gene mutations, and for copy number variation. Based on the results of these studies, KRT8 protein expression was measured in clinical ATC tumor specimens as well as in patient-derived xenograft (PDX) ATC cell lines. These cell lines were then also used to modulate KRT8 expression to determine the effects of the change in ATC cell survival. Finally, co-immunoprecipitation was used to identify potential KRT8 binding partners.

## 2. Results

### 2.1. Genomic Analysis

**Figure 1:**
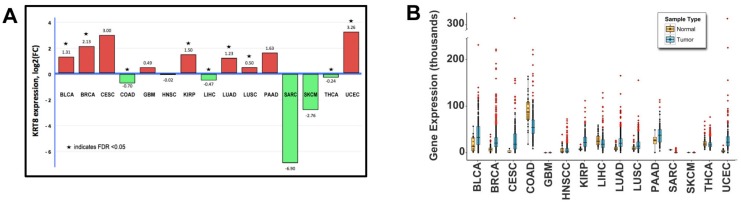

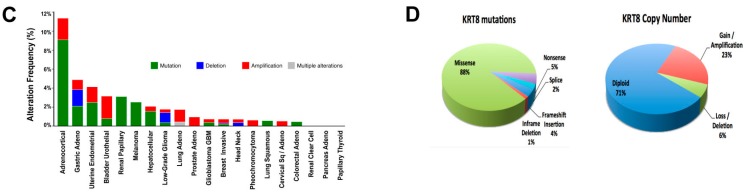
Keratin-8 (KRT8) genetic alterations are common in epithelial cancers. (**A**) *KRT8* expression by RNAseq for cancer versus normal in >3000 cases across 15 epithelial cancer types. Six cancer types showed a >2-fold increase in *KRT8* expression in cancer compared to normal, while the opposite was true for three cancer types; (**B**) *KRT8* expression plotted individually as dot-plots within each tumor type shows a population subset with elevated *KRT8* expression in most tumor types; (**C**) Genomic data from >5000 cancer patients were examined for *KRT8* alterations at the DNA level, which were present in up to 11% of cases depending on tumor type; (**D**) Among mutations, the majority were missense mutations (88%), while nonsense and deletion mutations were uncommon. Similarly, most copy number variations were due to amplification, and gene loss was less frequent.

Fifteen solid tumor types from the Cancer Genome Atlas (TCGA) with RNAseq gene expression data available were analyzed for *KRT8* expression patterns. Six epithelial tumor types had significantly elevated mean *KRT8* tumor expression compared to normal (false discovery rate (FDR) <0.05 for each), as summarized in Figure 1A. *KRT8* expression for each cancer case was plotted individually as dot-plots within each tumor type (Figure 1B) to examine expression levels in individual patients. These results show that within most cancer types, a subset of patients exhibit elevated *KRT8* expression. Only GBM, sarcoma, SKCM, and papillary thyroid cancer (PTC) showed no *KRT8*-elevation. Next, genomic datasets including 5713 patients across 20 solid cancer types were analyzed and visualized using Memorial Sloan Kettering’s web-based interface (cBioPortal.org; these results are summarized in Figure 1C,D). Genomic alterations in *KRT8* were present in 83 cases, ranging from 0 to 11% (mean 1.9%) of cases by cancer type, with the highest prevalence of *KRT8* genomic alterations observed in adrenocortical carcinoma (11.4%), stomach adenocarcinoma (4.9%), and uterine endometrial carcinoma (4.1%). Mutation was the most common genomic alteration (42/83; 51%), the majority of which were missense (88%). With respect to copy number variation affecting the *KRT8* gene, amplification was the most common event (23%), followed by gene loss (6%).

### 2.2. Patient Demographics and Keratin-8 (KRT8) Expression

**Figure 2:**
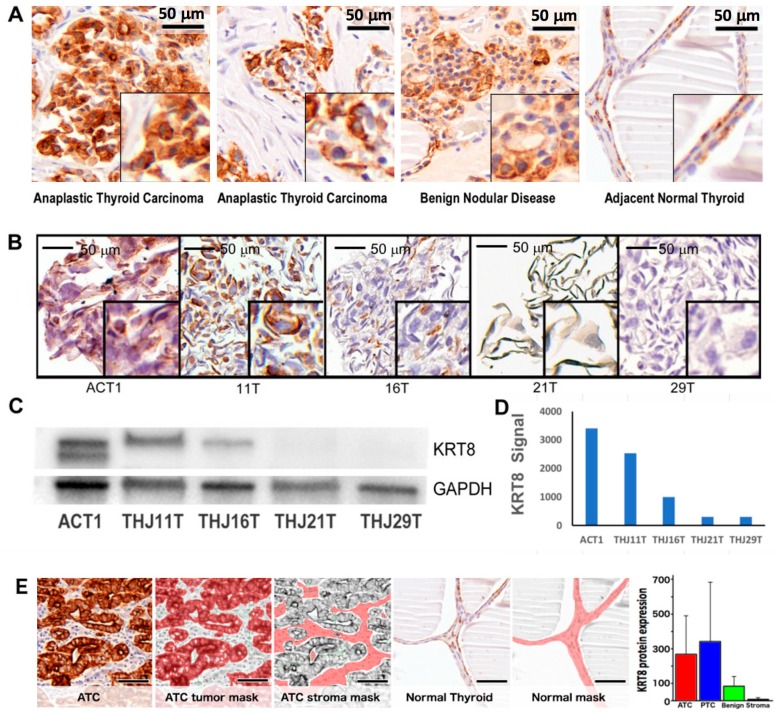
KRT8 is overexpressed in a subset of anaplastic thyroid carcinoma (ATC) tumors and cell lines. (**A**) There was elevated KRT8 expression in a subset of ATC tumors compared to normal thyroid and benign nodular disease by immunohistochemistry, and KRT8 expression was predominately cytoplasmic; (**B**) A similar heterogeneous KRT8 expression was observed in ATC patient-derived cell lines, with definitive expression in 3/5 and virtually undetectable expression in 2/5 ATC cell lines; (**C**) Immunoblot of lysates from these ATC cell lines using mouse monoclonal antibody specific for KRT8 demonstrated concordance with the immunohistochemistry data; (**D**) Semi-quantitative densitometry of these Western blot data, normalized for GAPDH and background signal; (**E**) Quantitative multispectral imaging of patient tumor and benign tissue (see methods) showed elevated KRT8 protein levels in ATC and papillary thyroid cancer (PTC) tumor tissue, compared to adjacent normal, tumor stroma, and benign nodular thyroid tissue. Scale bars in micrographs indicate 50 µm.

Next, KRT8 protein expression was measured in tumor and non-malignant patient samples (*n* = 17): eight patients with ATC, five with papillary thyroid cancer (PTC), and four with benign multinodular disease (BND). Patient characteristics are summarized in Table 1. KRT8 expression was determined quantitatively using mouse monoclonal antibody to KRT8 and nuance multispectral imaging methods. This is summarized in Table 2. Consistent with its known role as a cancer biomarker, KRT8 expression was elevated in a majority of malignant tissue samples (*n* = 13; mean nuance score 331.3 ± 266 standard deviation, SD) versus non-malignant tissue (*n* = 4; 83.8 ± 58 SD, *p* = 0.017). By cancer type, this was true for both ATC (*n* = 8; 268.2 ± 223 SD) and PTC (*n* = 5; 425.9 ± 332 SD) but was not statistically significant for either cancer separately versus normal, or ATC versus PTC. Subcellular distribution was predominately cytoplasmic for most samples (Figure 2A). Among ATC patient tumor samples, there was heterogeneous KRT8 expression, ranging from low (nuance score 29) to highly elevated (547). There were not enough cases to perform survival analysis.

### 2.3. KRT8 Expression in Cell Lines

KRT8 protein expression was determined in five patient-derived ATC cell lines by immunohistochemistry (IHC) and immunoblot. As shown in Figure 2B–D, a heterogeneous expression of KRT8 was observed among ATC cell lines, with 3/5 (60%) having definitive expression and 2/5 (40%) having virtually undetectable expression. Nuance quantitative expression analysis was concordant with immunoblot and IHC results (Table 2 and Figure 2E).

### 2.4. RNA-Interference-Mediated Knockdown of KRT8

**Figure 3:**
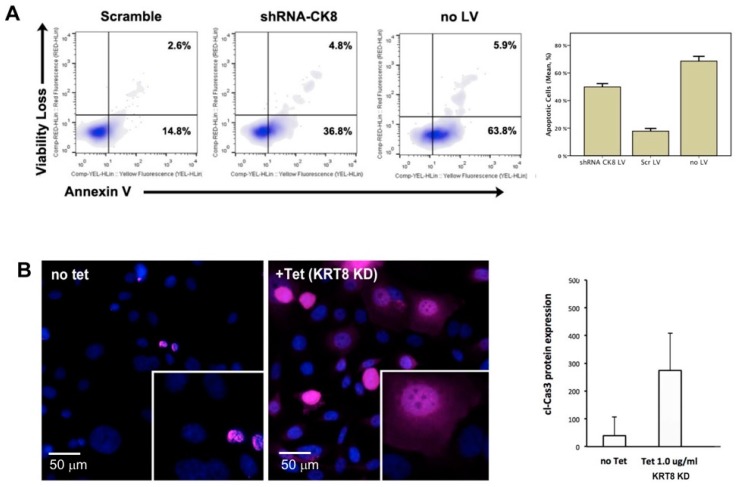
Lentiviral shRNA-mediated *KRT8* knockdown results in increased apoptosis in ATC cells. Controls included no LV (thus puromycin sensitive), and scrambled shRNA (no *KRT8* targeting, but with the puromycin resistance gene). (**A**) Cell count, viability, and apoptosis were measured by flow cytometry using annexin-V and propidium iodide staining. Shown is a summary scatter plot combining all replicate runs with identical gating. There was a right-shifted annexin-V population (consistent with an increase in apoptotic cells) at 72 h following lentivirus transduction of ACT1 (KRT8 expressing) cells with KRT8-targetted shRNA; (**B**) Cleaved caspase-3 (cCas3) immunohistochemistry of ACT1 cells, with shRNA knockdown using a tetracycline inducible lentiviral shRNA construct. There is an increase in cCas3 expression in ACT1 cells 48 h following addition of 1.0 µg/mL tetracycline, compared to no-tetracycline controls.

To test the hypothesis that KRT8 plays a functional role in ATC, replication incompetent lentivirus was used to stably express an shRNA specific for *KRT8* in ACT1 cell line (KRT8 expressing). This resulted in 90% reduction in *KRT8* mRNA expression as determined by rt-PCR (Supplementary Materials, Figure S1A). Following transduction, cells were maintained in media with 4 µg/mL puromycin for 72 h. Cells were then analyzed for viability and apoptosis using a flow cytometry-based apoptosis assay for annexin-V (Cell Viability and Apoptosis Kit, EMD Millipore). Controls included ACT1 cells transduced with nonsense scramble shRNA encoding lentivirus (with puromycin resistance gene), and wild type ACT1 cells without lentivirus (and thus lacking puromycin resistance). Both cells subjected to RNAi-mediated *KRT8* knockdown, and untreated controls (lacking the puromycin resistance gene) exhibited a decrease in the number of viable, non-apoptotic cells recovered (mean 253.2 ± SD 10.6 cells/µL and 300.4 ± 13.6 cells/µL respectively) compared to scramble controls (1038.6 ± 53.7 cells/µL, *p* < 0.001 for each). This loss of viability was partially mediated by increased apoptosis, with 49.9% ± SD 2.4 of KRT8 knockdown cells in early or late apoptosis versus 17.8% ± 2.0 of scramble-shRNA control cells, *p* < 0.001). Flow cytometry results are summarized in Figure 3A and Supplementary Materials, Figure S1B,C.

### 2.5. Tet-Inducible KRT8 Knockdown and Apoptosis

Given the substantial deleterious effect on cell survival following *KRT8* knockdown, a tetracycline-inducible shRNA lentiviral construct was created (see Methods). This model was used to further interrogate *KRT8* knockdown effects in ACT1^+Tetr/ck8shRNA#c^ cells by immunohistochemistry for cleaved caspase-3 (cCas3). Cells grown with tetracycline 1.0 µg/mL resulted in 80% reduction in *KRT8* gene expression compared to the scrambled control (Supplementary Materials). At 48 h, there was an elevation in cCas3 expression in knockdown (nuance score 274.2 ± 135 SD) compared to no-tetracycline controls (39.3 ± 69 SD, *p* < 0.0001), shown in Figure 3B. In the no-Tet condition, the cells that do express cCas3 only display nuclear localization, compared to both cytoplasmic and nuclear cCas3 in the +Tet (KRT8 knockdown) cells.

### 2.6. KRT8 Overexpression

**Figure 4:**
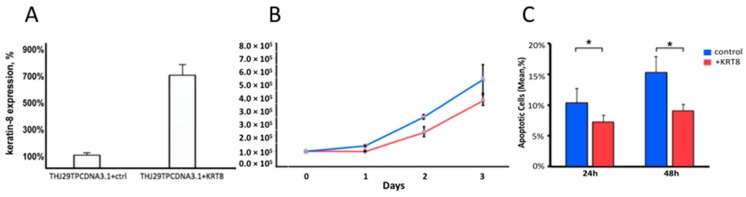
KRT8 expression decreases cell viability but confers resistance to apoptosis under redox stress conditions in the THJ29T cell line (KRT8 non-expressing ATC). (**A**) rt-PCR confirmation of KRT8 expression in THJ29T^PCDNA3.1+KRT8^ compared to THJ29T^PCDNA3.1+scr^; (**B**) Cell viability was inhibited following KRT8 plasmid transduction; (**C**) Under redox stress conditions (media with 100 µM hydrogen peroxide), there was a decrease in apoptotic cells in THJ29T^PCDNA3.1+KRT8^ cells, compared to scramble control. * indicates result is significant at *p* < 0.05.

To investigate the effects of KRT8 forced overexpression, THJ29T (KRT8 low-expressing) cells were transduced with PCDNA3.1 plasmid, incorporating either the scrambled nonsense gene (THJ29T^PCDNA3.1+Scr^) or *KRT8* gene (THJ29T^PCDNA3.1+KRT8^). KRT8 expression was confirmed by rt-PCR (Figure 4A) and immunoblot. Following selection in antibiotic media, clonal sub-cultures of cells were plated at controlled density in replicate 6-well plates and cell viability was measured at 24, 48, and 72 h. THJ29T^PCDNA3.1+KRT8^ cells exhibited lower cell viability compared to control (Figure 4B). There was no difference in apoptosis at 24 or 48 h post transduction; however, when experiments were repeated under redox stress conditions (adding 100 μM hydrogen peroxide to media), KRT8 overexpression was associated with reduction in apoptosis, with 15.3% ± 1.3 SD of THJ29T^PCDNA3.1+Scr^ cells being apoptotic versus 9.2% ± 1.6 THJ29T^PCDNA3.1+KRT8^ cells at 48 h (Figure 4C, *p* = 0.006).

### 2.7. Identification of Annexin-A2 as a KRT8 Binding Protein

**Figure 5:**
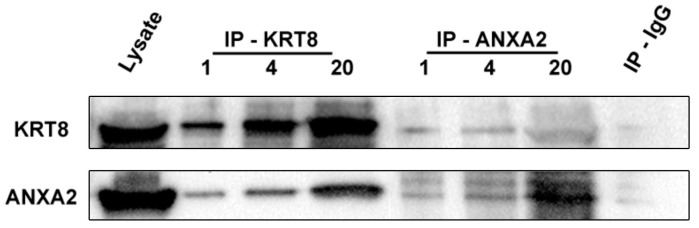
Magnetic bead-based immunoprecipitation was performed on ACT1 lysates (see Methods) using KRT8 (IP-KRT8 group), annexin-A2 (IP-ANXA2 group), and non-targeting mouse IgG (IP-IgG) antibodies. Immunoprecipitation eluates (at increasing loading amounts) and unprocessed ACT1 lysate (positive control) were separated by polyacrylamide gel and immunoblot performed with KRT8 and annexin-A2 antibodies.

Whole-cell lysates of ACT1 were probed with anti-KRT8 antibody, covalently bonded to magnetic beads and subsequently eluted as described in Methods. The ATC cell line ACT1 was chosen for these studies based on its elevated KRT8 expression and rapid growth characteristics. The immunoprecipitation eluate was then subjected to PAGE/HPLC/tandem mass spectrometry to identify potential binding proteins. Several previously confirmed KRT8 binding partners were identified by sequence analysis, including keratin-18, fibronectin, and periplakin (Supplementary Materials, Table S3). In addition, annexin-A2 also was identified as a putative binding partner, consistent with existing reports identifying possible interactions of other keratins with annexin family members [16,17,18]. Annexin-A2 was identified in 4/7 MS/MS samples, with a median of 11 unique spectra (range 8–43) resulting in direct identification of mean 25.8% of the protein (range 6.8–54). Using Peptide Prophet, the protein identification probability for annexin-A2 was >99.99% for each sample. Next, co-immunoprecipitation was used to further query these protein–protein interactions. Magnetic bead-based immunoprecipitation, using antibodies to KRT8, annexin-A2, and non-targeting mouse IgG, was performed on ACT1 lysates. Immuno-precipitation eluates (at increasing loading amounts) and unprocessed ACT1 lysate (positive control) were separated by polyacrylamide gel and probed by Western blot with KRT8 and annexin-A2 antibodies. KRT8 and annexin-A2 were demonstrated in both KRT8 and annexin-A2 immunoprecipitation eluate, and not in the control IgG eluate (Figure 5).

## 3. Discussion

We provide evidence that KRT8 is significantly elevated in a subset of patients with various types of cancers, including in ATC patient tumors and tumor-derived ATC PDX cell lines. KRT8 knockdown and overexpression, using ATC cell lines, further implicate KRT8 in ATC cell growth and resistance to apoptosis. It is also interesting to note that following KRT8 knockdown, there is not only an increase in number of cells expressing cCas3, but also a change in the subcellular localization of cCas3. Caspase-3 has been shown to exert its apoptotic effects via interaction with both nuclear and cytoplasmic target proteins. It is possible that KRT8 may be affecting the subcellular localization of cCas3, although the mechanism for this is not clear. While previous reports have suggested that KRT8 may play a role in either promoting or inhibiting the aggressive behavior of some cancers, this is the first report providing evidence that KRT8 is an important driver of ATC tumor cell survival. As such, these data provide preliminary support for the concept that KRT8 may be useful as a novel therapeutic target in ATC. 

While the known role for keratins (including KRT8) is that of a structural cytoskeletal protein [5], there have been studies suggesting more complex non-mechanical functions for KRT8 as well as other keratins. For example, Gilbert et al. have shown in non-malignant liver cells that keratin 8/18 likely plays a functional role in protecting hepatocytes from apoptosis [19]. Conversely, KRT8/18 depletion results in increased hepatocellular and cervical cancer cell migration and invasion in vitro, while simultaneously sensitizing these cells to cisplatin-induced apoptosis [20]. In vitro, KRT8 can be found localized to the cell membrane and interacts with human leukocyte antigen class I, potentially modulating immune cell interaction [21]. Keratin-17 has been implicated in skin cancer progression by modulating the TH1–TH2 T-cell phenotype balance [22] and also has been shown to play a role in cervical squamous cell tumor biology by binding to and exporting the tumor suppressor p27^KIP1^ from the nucleus. Thus, the concept of a non-mechanical role for KRT8 in ATC tumor etiology is not without precedent. In fact, many cytoskeletal proteins (notably β-catenin, vimentin, actin, tubulin, and lens crystallins [9,23,24,25]) are now known to play dual roles as both structural proteins and as intracellular signaling proteins. These may provide exemplar models upon which to base future mechanistic studies of KRT8 (and other keratins) non-mechanical functions. Our studies provide evidence of reciprocal binding between annexin-A2 and KRT8. This is potentially important, as annexin-A2 has known interactions with both the redox and apoptosis pathways. Annexin-A2 has been shown to act in some cancers as a redox sink for peroxide molecules [26], thus allowing rapid detoxification of peroxide intermediates generated by the elevated metabolic rate of most cancer cells. It is also regulated by reversible glutathionylation, and may mediate free radical and radiation-induced apoptosis [27,28]. Interestingly, annexin-A2 also has been implicated in gemcitabine resistance in pancreatic cancer via the AKT/mTOR pathway [29]. One of the defining clinical features of anaplastic thyroid carcinoma is its resistance to traditional chemotherapeutic agents [3]. KRT8/annexin-A2 may partially mediate this resistance and could be a potential target for the development of new therapeutic strategies.

## 4. Material and Methods

### 4.1. Human Patient Cohort Selection

Patient information and sample collection was approved for this study by the Augusta University Institutional Review Board (HAC06-07-012 “Molecular Analysis of Benign and Malignant Thyroid Neoplasms, PI: Weinberger, P; 23 July 2010—30 June 2016). Consecutive patients with a histologic diagnosis of ATC from 2003 to 2013 at Augusta University were included, as well as a convenience sampling of patients with papillary thyroid cancer (PTC) from the same time frame. Exclusion criteria were lack of available FFPE archival specimen.

### 4.2. General Laboratory Reagents

All reagents and chemicals used were purchased from Fisher Scientific (Hampton, NH, USA) unless otherwise specified below. PDX and ATC patient-derived cell lines: Five well–characterized anaplastic thyroid cancer cell lines, four derived as xenografts from patient ATC primary tumors, were obtained as gifts from Dr. J. Copland (Mayo Clinic, Jacksonville FL; THJ11T, THJ16T, THJ21T, and THJ29T) [30] and one immortalized ATC line was obtained as a gift from Dr. S. Ohata (Tokushima University, Tokushima, Japan; ACT1) [31]. All cell line identities were independently confirmed in our laboratory by short tandem repeat (STR) DNA analysis before experimental use (Supplementary Materials, Table S1).

### 4.3. Genomic Data Analysis

The Cancer Genome Atlas (TCGA) RSEM-normalized [32] RNA sequencing data for all primary tumor and tissue normal samples from 15 solid-tumor were downloaded using TCGA-Assembler [33] and log-transformed. Genomic alterations in KRT8 from additional patient cohorts were summarized by cancer type and visualized using the cBioPortal.org interface.

### 4.4. Statistical Analysis

Flow cytometry data were converted to FCS format and visualized in FlowJo v9 (FlowJo LLC, Ashland, OR, USA). Genomic data were analyzed using LIMMA [34] package in a custom R script. All other statistical analyses were performed using SPSS version 23.0 (IBM Corporation, Carey, NC, USA) and were two-tailed where appropriate. A *p* value of 0.05 was set for determining significance. Graphs for data visualization were produced using SPSS, MS Excel for Mac 14.4 (Microsoft, Redmond, WA, USA), LIMMA, and FlowJo.

### 4.5. Immunohistochemistry

Cell lines for paraffin embedding and tissue microarray (TMA) construction methods were as previously described [35], with antigen retrieval performed using Diva Decloaker (Biocare Medical, Concord, CA, USA) for 1 minute in a 100 °C pressure cooker. Primary antibody incubation was at 4 °C overnight: KRT8 (mouse monoclonal, clone 4.1.18; Millipore, Billerica, MA, USA, 1:150 dilution); cleaved caspase-3 (cCas3, mouse monoclonal, Abcam, Cambridge, MA, USA, 1:150 dilution). The secondary antibody was horseradish peroxidase conjugated goat anti-mouse antibody (Envision; DAKO North America, Carpinteria, CA, USA), followed by diaminobenzidine (DAB) chromagen for visualization of KRT8. For cCas3, 20 nM goat anti-mouse antibody conjugated to 605 nM quantum dot (Invitrogen, Carlsbad, CA, USA) and DAPI nuclear counterstain were used.

### 4.6. Quantitative In-Situ Protein Expression Measurement

Protein expression and TUNEL quantitative analysis were determined using a multispectral imaging workstation (Nuance FX, Perkin Elmer, Akron, OH, USA) attached to a Zeiss Axiophot I microscope (Carl Zeiss Microscopy, Thornwood, NY, USA). Images were acquired at 40× magnification and spectrally unmixed to individual dye channels based on the spectral libraries, allowing quantitative measurement of protein expression independent of other signal intensities [36,37], transformed to an expression scale from 1 (lowest) to 1000 (highest). For TUNEL assay quantitation, co-localization of the DAPI and Br-dUTP signal was determined on a per-pixel basis and expressed as a percentage.

### 4.7. shRNA Lentivirus Knockdown

Commercially available replication-incompetent lentivirus shRNA constructs targeting the KRT8 gene, which encodes the *KRT8* protein, and a puromycin resistance gene were purchased (sc-35156-V, SantaCruz Biotechnology, La Jolla, CA, USA) and used as per the manufacturer’s instructions. Negative controls consisted of identical lentiviral particles, but encoding a nonsense shRNA sequence (SantaCruz Biotechnology). Knockdown efficiency was determined by RT-PCR for KRT8 mRNA.

### 4.8. Tetracycline-Inducible shRNA Knockdown

Because of the severe decrease in cell viability following KRT8 knockdown, stable clones were not able to be selected and propagated for further study. Therefore, an inducible KRT8 knockdown ATC line (designated ACT1^+tetR/+CK8shRNA#C^) was created by co-transduction of ATC cell lines with lentiviral particles containing the TetR regulatory element (GenTarget, San Diego CA, USA), and a tetracycline-inducible shRNA lentiviral construct. Stable double-transduction clones were selected in DMEM + 10% certified tetracycline-free FBS, neomycin and puromycin, and confirmed by RT-PCR and Western blot. 

### 4.9. Flow-Cytometry-Based Cell Viability and Apoptosis Assays

Cells were seeded in 12-well tissue culture plates (Corning Life Sciences, Corning, NY, USA) at 30% confluence. Twenty-four hours later (at 50% confluence), lentiviral transduction (SantaCruz sc-35156-V) was performed as described above at MOI = 1. Following media exchange to selection media, cells were allowed to grow for 48 or 96 h followed by trypsin release and analysis. A commercially available apoptosis and cell viability flow-cytometry-based assay was used as per the manufacturer’s recommendations (Annexin V/Apoptosis Muse Assay, EMD Millipore, Billerica, MA, USA) performed on a benchtop flow cytometer (Muse, EMD Millipore).

### 4.10. Western Blot

Adherent cells were lysed with modified RIPA buffer with phosphatase and protease inhibitors (PhosSTOP and cOmplete Ultra, Roche Life Sciences, Indianapolis, IN, USA). Standard Western blot under reducing conditions was performed, with a 3% bovine serum albumin block after transfer to PVDF membrane. The membranes were probed with primary antibodies (Supplementary Materials, Table S2), then washed in Tris-Buffered Saline with 0.05% Tween-20 (TBST) and incubated with goat-anti rabbit or goat-anti mouse HRP-conjugated (Envision, Dako) overnight at 4 °C. Membranes were washed in TBST and then incubated with SuperSignal West chemiluminescent substrate (Thermo Scientific, Waltham, MA, USA) and imaged.

A fluorescent apoptosis TUNEL assay (Abcam) was used to quantify fragmented DNA. Imaging was performed using the Nuance MSI system at 40× and a triple-emission fluorescent filter set (Chroma, Irvine, CA, USA). Nuance image cubes were captured at 20 nM wavelength intervals from 460 to 720 nM.

### 4.11. KRT8 Plasmid Transduction

The construction of a KRT8 expression plasmid has been previously described [21]. THJ29T cells (normally low-KRT8 expressing) were transduced with PCDNA3.1 plasmid, plus either scrambled cDNA or the full-length KRT8 coding sequence. Stable clones were selected, propagated, and mRNA and protein harvested to confirm KRT8 overexpression using rt-PCR and Western blot for KRT8 as above. These cells (designated THJ29T^PCDNA3.1+Scr^ or THJ29T^PCDNA3.1+KRT8^) were analyzed as above for apoptosis and cell proliferation, both at standard conditions and under redox stress conditions, by adding 100 μM hydrogen peroxide to standard media for 24 h.

### 4.12. Mass Spectrometry

Following immunoprecipitation, IP product was separated on polyacrylamide gels into 3 size distribution regions, followed by in-gel tryptic digestion and elution with 50% acetonitrile with 0.1% trifluoroacetic acid. Samples were then subjected to tandem mass spectrometry peptide sequencing experiments, as previously described [38]. Five tandem mass spectra were collected in a data-dependent manner following each survey scan.

### 4.13. Protein Identification

Tandem mass spectra were analyzed using Mascot (Matrix Science, London, UK; version 2.2.04) and Sequest (Thermo Fisher Scientific, San Jose, CA, USA; version SRF v. 3) and Scaffold (version 4.3.4, Proteome Software Inc., Portland, OR, USA) to catalogue MS/MS based peptide and protein identifications. Peptide and protein identification parameters were set to result in a 0.3% false discovery rate (FDR) for peptide mapping and a 0.1% FDR for protein identification.

### 4.14. Co-Immunoprecipitation

Magnetic bead-based immunoprecipitation reagents were either purchased commercially (KRT8; PA1240-M, Antagene Inc., Santa Clara, CA, USA) or prepared in-house. Annexin A2 and control IgG magnetic beads were prepared using Pierce Protein A/G coupled magnetic beads (Thermo-Fisher Scientific, Waltham, MA, USA) according to the manufacturer’s recommended protocol. Monoclonal rabbit antibody to Annexin A2 (RabMAb clone EPR13052, Abcam) or non-immune rabbit IgG fraction (ab27478, Abcam) was purified to remove BSA and preservative using MelonGel spin columns (Thermo-Fisher Scientific), followed by incubation with the magnetic beads containing pre-bound Protein-A/G. Co-immunoprecipitation experiments were repeated in triplicate.

## 5. Conclusions

In summary, KRT8 expression is elevated in a subset of ATC tumors. Elevated KRT8 expression is important for ATC tumor cell survival, since, as demonstrated in this study, siRNA-mediated knockdown of KRT8 expression results in increased apoptosis and loss of cell viability in ATC-derived cells in vitro; conversely, expression of KRT8 in normally low-expressing ATC cells confers resistance to apoptosis under conditions of redox stress. Preliminary results suggest that KRT8 interacts with annexin-A2, a protein known to mediate redox and free-radical-induced apoptosis. We propose, therefore, that KRT8 is not only a diagnostic cancer biomarker, but also may be involved in ATC cell survival. As such, the role of KRT8 and annexin-A2-mediated survival signaling in ATC warrants further scrutiny as a potential novel therapeutic target in ATC patients.

## Figures and Tables

**Table 1:** Summary of patient characteristics.

	All Patients	ATC	PTC	BND
Number (*n*)	17	8	5	4
Age, range (mean), years	37–82 (60)	37–82 (65)	38–78 (56)	48–59 (54)
Gender (male/female)	5/12	3/5	1/4	1/3
Ethnicity				
African-American	7	1	3	3
Caucasian	10	7	2	1
Asian or other	0	0	0	0

ATC = anaplastic thyroid carcinoma; PTC = papillary thyroid carcinoma; BND = benign nodular disease.

**Table 2:** Keratin-8 quantitative expression by multispectral imaging.

	Keratin-8 Expression (Mean ± SD)	*p* Value (Compared to Benign)
Cancer Tissue	331.3 ± 266	0.017 *
ATC	268.2 ± 223	0.100
PTC	425.9 ± 332	0.131
Benign Thyroid	83.8 ± 58	–
BND	93.5 ± 43	0.82
Adjacent Normal	80.0 ± 67	0.92
Stroma	9.4 ± 8	0.013 *
ACT1 cell line	490.3 ± 178	–
THJ11T cell line	138.2 ± 46	–
THJ16T cell line	162.7 ± 51	–
THJ29T cell line	1.40 ± 1.2	–

* = significant at *p* < 0.05. ATC = anaplastic thyroid carcinoma; PTC = papillary thyroid carcinoma; BND = benign nodular disease.

## Supplementary Materials

Supplementary materials can be found at http://www.mdpi.com/1422-0067/19/2/577/s1.
