# Supplementary material for: Cytokeratin-8 in Anaplastic Thyroid Carcinoma: More Than a Simple Structural Cytoskeletal Protein
Source: Int J Mol Sci. 2018 Feb 14;19(2):577. doi: 10.3390/ijms19020577 (PMC5855799; doi:10.3390/ijms19020577)
Supplement: Supplementary file 1 [file ijms-19-00577-s001.pdf]

Supplementary Materials

Table S1. 1 STR analysis of cell lines. To minimize the chance of inadvertent cell line misidentification, STR analysis is performed annually on cell lines in use and results compared to published data (when available), or previous STR data.

Cell Line	Source	Experimental STR	Reference STR	Source for Comparison STR
THJ11T	ATC patient xenograft<br><br>J.Copland; Mayo Clinic – Jacksonville[1]	Amelogenin: XY<br>D5S818: 9,13<br>D13S317: 8,13<br>D7S820: 9<br>**D16S539: 12,4**<br>vWA: -<br>TH01: 5,6<br>TPOX: 8,11<br>CSF1PO: 12<br>D18S51: 12,14<br>D3S1358: 14,17<br>D8S1179: 13<br>FGA: 21,24	Amelogenin: XY<br>D5S818: 9,13<br>D13S317: 8,13<br>D7S820: 9<br>**D16S539: 12,13**<br>vWA: -<br>TH01: 5,6<br>TPOX: 8,11<br>CSF1PO: 12<br>D18S51: 12,14<br>D3S1358: 14,17<br>D8S1179: 13<br>FGA: 21,24	[1]
THJ16T	ATC patient xenograft<br><br>J.Copland; Mayo Clinic – Jacksonville[1]	Amelogenin: XX<br>D5S818: 11,12<br>D13S317: -<br>D7S820: 10,12<br>D16S539: 6,12<br>vWA: 16,17<br>TH01: 8,9<br>TPOX: 8,11<br>CSF1PO: 11,14<br>D18S51: 17,19<br>D3S1358: 15,16<br>D8S1179: 12,13<br>FGA: 23,27	Amelogenin: XX<br>D5S818: 11,12<br>D13S317: 10,11<br>D7S820: 10,12<br>D16S539: 6,12<br>vWA: 16,17<br>TH01: 8,9<br>TPOX: 8,11<br>CSF1PO: 11,14<br>D18S51: 17,19<br>D3S1358: 15,16<br>D8S1179: 12,13<br>FGA: 23,27	[1]
THJ21T	ATC patient xenograft<br><br>J.Copland; Mayo Clinic – Jacksonville[1]	Amelogenin: XY<br>D5S818: 12,13<br>D13S317: 11,12<br>D7S820: 8,10<br>D16S539: 11,12<br>vWA: 16,17<br>TH01: 6<br>TPOX: 8,12<br>CSF1PO: 11,15<br>D18S51: 13,15<br>D3S1358: 15,17<br>D8S1179: 9,14<br>FGA: 24	Amelogenin: XY<br>D5S818: 12,13<br>D13S317: 11,12<br>D7S820: 8,10<br>D16S539: 11,12<br>vWA: 16,17<br>TH01: 6<br>TPOX: 8,12<br>CSF1PO: 11,15<br>D18S51: 13,15<br>D3S1358: 15,17<br>D8S1179: 9,14<br>FGA: 24	[1]
ACT1	S. Ohata; Tokushima University[2]	Amelogenin: XX<br>D5S818: 11,13<br>D13S317: 10, 12<br>D7S820: 10, 11<br>D16S539: -<br>vWA: 17, 18<br>TH01: 6, 9<br>TPOX: 8, 8<br>CSF1PO: 11, 12<br>D18S51: 15, 16<br>D3S1358: 16, 16<br>D8S1179: 11, 14<br>FGA: 23, 24	Reference STR signature undefined	Reference STR signature undefined

BOLD indicates discrepancy between reference and experimental STR typing. - indicates data not available.

Table S2. Antibodies for western blot, immunohistochemistry.

Antibody	Species/Clonality	Designation or Clone	Dilution(s)	Manufacturer
Keratin 8	Mouse monoclonal	C51	1:1000 (WB)	Invitrogen
Keratin 8	Mouse monoclonal	4.1.18	1:50 (IHC)<br>1:1000 (WB)	EMD Millipore
Pan-cytokeratin	Mouse monoclonal (cocktail)	AE1 / AE3	1:200 (IHC)	Abcam
Glyeraldehyde 3-phosphate dehydrogenase (GAPDH)	Mouse monoclonal	9484	1:5000 (WB)	Abcam
TetR Protein	Rabbit Polyclonal		1:1000 (WB)	Sigma Aldrich
Cleaved caspase-3	Mouse monoclonal		1:150 (IHC)	Abcam

Table S3. Top 50 proteins (by total spectral count).

Identified Protein ∣ Gene	Molecular Weight	Gel 25-37 kDa	Gel 37-50 kDa	Gel 37-50 kDa	Gel 50-150 kDa	Gel 150-250 kDa	Gel 150-250 kDa	Gel 250+ kDa	Total Spectral Counts
Myosin-9 ∣ Gene:MYH9	227 kDa	0	0	1	0	101	682	82	866
Plectin ∣ Gene:PLEC	532 kDa	0	0	2	0	268	40	373	683
**Keratin, type II cytoskeletal 8 ∣ Gene:KRT8**	**54 kDa**	**17**	**159**	**141**	**55**	**45**	**19**	**21**	**457**
Keratin, type I cytoskeletal 19 ∣ Gene:KRT19	44 kDa	31	162	149	34	24	15	15	430
Keratin, type II cytoskeletal 7 ∣ Gene:KRT7	51 kDa	17	91	95	33	30	12	14	292
Keratin, type II cytoskeletal 1 ∣ Gene:KRT1	66 kDa	23	44	30	53	61	57	21	289
**Keratin, type I cytoskeletal 18 ∣ Gene:KRT18**	**48 kDa**	**10**	**102**	**82**	**26**	**14**	**5**	**7**	**246**
DNA-dependent protein kinase catalytic subunit ∣ Gene:PRKDC	469 kDa	0	0	0	0	116	33	72	221
Keratin, type I cytoskeletal 17 ∣ Gene:KRT17	48 kDa	9	68	54	22	50	8	7	218
Myosin-10 ∣ Gene:MYH10	229 kDa	0	0	0	0	16	187	11	214
Keratin, type II cytoskeletal 2 epidermal ∣ Gene:KRT2	65 kDa	14	30	21	38	48	47	13	211
Keratin, type I cytoskeletal 10 ∣ Gene:KRT10	59 kDa	13	27	20	39	42	39	11	191
Keratin, type I cytoskeletal 14 ∣ Gene:KRT14	52 kDa	9	46	48	24	44	14	5	190
Actin, cytoplasmic 2 ∣ Gene:ACTG1	42 kDa	15	71	58	13	16	8	3	184
Keratin, type II cytoskeletal 5 ∣ Gene:KRT5	62 kDa	5	21	26	43	46	21	8	170
Cytoplasmic dynein 1 heavy chain 1 ∣ Gene:DYNC1H1	532 kDa	0	0	0	0	101	0	58	159
Keratin, type I cytoskeletal 16 ∣ Gene:KRT16	51 kDa	6	32	36	20	38	15	0	147
Keratin, type II cytoskeletal 6A ∣ Gene:KRT6A	60 kDa	5	22	24	29	41	24	0	145
Keratin, type I cytoskeletal 9 ∣ Gene:KRT9	62 kDa	13	16	12	34	33	31	6	145
Desmoplakin ∣ Gene:DSP	332 kDa	0	0	1	3	66	26	9	105
Alpha-actinin-4 ∣ Gene:ACTN4	105 kDa	0	0	0	84	15	0	0	99
Keratin, type I cytoskeletal 13 ∣ Gene:KRT13	50 kDa	7	34	32	0	10	12	0	95
Tubulin beta chain ∣ Gene:TUBB	50 kDa	9	41	19	13	12	0	0	94
Actin, alpha cardiac muscle 1 ∣ Gene:ACTC1	42 kDa	8	37	30	8	9	0	0	92
Keratin, type II cytoskeletal 6B ∣ Gene:KRT6B	60 kDa	0	0	0	26	40	24	0	90
DNA topoisomerase 1 ∣ Gene:TOP1	91 kDa	2	3	4	56	15	5	4	89
Nuclear mitotic apparatus protein 1 ∣ Gene:NUMA1	238 kDa	0	0	0	0	50	39	0	89
Pyruvate kinase isozymes M1/M2 ∣ Gene:PKM	58 kDa	3	5	1	52	23	3	1	88
Tubulin beta-4B chain ∣ Gene:TUBB4B	50 kDa	7	40	18	12	11	0	0	88
Filamin-A ∣ Gene:FLNA	281 kDa	0	0	0	0	78	4	0	82
Ras GTPase-activating-like protein IQGAP1 ∣ Gene:IQGAP1	189 kDa	1	0	0	1	62	16	0	80
Alpha-actinin-1 ∣ Gene:ACTN1	103 kDa	0	0	0	66	8	0	0	74
Clathrin heavy chain 1 ∣ Gene:CLTC	192 kDa	0	0	0	1	70	0	0	71
Filamin-B ∣ Gene:FLNB	278 kDa	0	0	0	0	70	0	0	70
Annexin A2 ∣ Gene:ANXA2	39 kDa	43	8	0	8	11	0	0	70
Neuroblast differentiation-associated protein ∣ Gene:AHNAK	629 kDa	0	0	0	1	64	0	4	69
Myosin-14 ∣ Gene:MYH14	228 kDa	0	0	0	0	19	43	7	69

Alpha-enolase ǀ Gene:ENO1	47 kDa	6	35	14	7	4	0	0	66
Keratin, type II cytoskeletal 6C ǀ Gene:KRT6C	60 kDa	0	0	0	0	42	23	0	65
Tubulin alpha-1C chain ǀ Gene:TUBA1C	50 kDa	2	34	17	4	4	1	0	62
Keratin, type I cytoskeletal 15 ǀ Gene:KRT15	49 kDa	0	23	24	0	10	0	0	57
**Fibronectin ǀ Gene:FN1**	**263 kDa**	**0**	**0**	**0**	**0**	**34**	**15**	**7**	**56**
Elongation factor 1-alpha 1 ǀ Gene:EEF1A1	50 kDa	4	19	10	6	11	4	1	55
Spectrin alpha chain, non-erythrocytic 1 ǀ Gene:SPTAN1	285 kDa	0	0	1	0	52	0	0	53
POTE ankyrin domain family member E ǀ Gene:POTEE	121 kDa	0	27	25	0	0	0	0	52
Histone H4 ǀ Gene:HIST1H4A	11 kDa	32	2	2	3	5	1	3	48
**Periplakin ǀ Gene:PPL**	**205 kDa**	**0**	**0**	**0**	**0**	**25**	**23**	**0**	**48**
Poly(U)-binding-splicing factor PUF60 ǀ Gene:PUF60	60 kDa	2	6	4	31	4	0	0	47
Ribosome-binding protein 1 ǀ Gene:RRBP1	152 kDa	0	0	0	11	31	5	0	47

Replicate ACT1 cell lysates were probed by anti-KRT8 magnetic bead immunoprecipitation followed by polyacrylamide gel electrophoresis separation. Approximate size distributions (from adjacent marker lanes) were excised, subjected to tryptic in-gel digestion, purification and LC/MS-MS analysis. Existing experimentally proven binding partners of keratin 8 are indicated in **bold**.

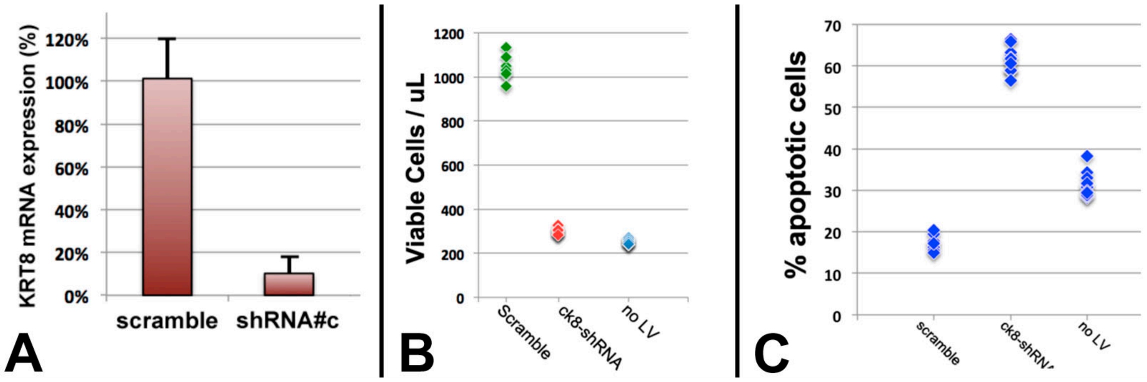

Figure S1. KRT8 RNA-i lentiviral knockdown. (**A**) Six shRNA targeting KRT8 were tested (data not shown), shRNA#c was chosen for subsequent experiments and resulted in 90% reduction in KRT8 mRNA expression compared to scrambled shRNA lentivirus; (**B**) A flow cytometry based assay (Muse Annexin V/Dead Cell Kit) to determine cell viability and apoptosis status. Both no-lentivirus control (lacking puromycin resistance gene) and KRT8 knockdown, resulted in decreased number of viable cells; (C) Similarly, there was an increase in apoptotic cells in both no-lentivirus and KRT8 knockdown cells.

References

1. Marlow, L.A.; D'Innocenzi, J.; Zhang, Y.; Rohl, S.D.; Cooper, S.J.; Sebo, T.; Grant, C.; McIver, B.; Kasperbauer, J.L.; Wadsworth, J,T.; et al. Detailed molecular fingerprinting of four new anaplastic thyroid carcinoma cell lines and their use for verification of RhoB as a molecular therapeutic target. *J. Clin. Endocrinol. Metab.* **2010**, *95*, 5338–5347.
2. Chung, S.H.; Onoda, N.; Ishikawa, T.; Ogisawa, K.; Takenaka, C.; Yano, Y.; Hato, F.; Hirakawa, K. Peroxisome proliferator-activated receptor γ activation induces cell cycle arrest via the p53-independent pathway in human anaplastic thyroid cancer cells. *Jpn. J. Cancer Res.* **2002**, *93*, 1358–1365.
